# Long-Term Trends (1994–2011) and Predictors of Total Alcohol and Alcoholic Beverages Consumption: The EPIC Greece Cohort

**DOI:** 10.3390/nu13093077

**Published:** 2021-09-01

**Authors:** Nikolaos Skourlis, Paraskevi Massara, Ioannis Patsis, Eleni Peppa, Klea Katsouyanni, Antonia Trichopoulou

**Affiliations:** 1 Hellenic Health Foundation, Kaisareias 13 & Alexandroupoleos, 11527 Athens, Greece; massaraevi@yahoo.com (P.M.); ptsyannis@gmail.com (I.P.); e.peppa@hhf-greece.gr (E.P.); kkatsouy@med.uoa.gr (K.K.); atrichopoulou@hhf-greece.gr (A.T.); 2 Department of Hygiene Epidemiology and Medical Statistics, Medical School, National and Kapodistrian University of Athens, Mikras Asias 75, 11527 Athens, Greece

**Keywords:** alcohol consumption, longitudinal, EPIC, linear mixed models

## Abstract

The aim of this study was to evaluate the longitudinal changes in alcohol consumption (total alcohol and types of alcoholic beverages) of the Greek EPIC cohort participants (28,572) during a 17-year period (1994–2011), with alcohol information being recorded repeatedly over time. Descriptive statistics were used to show crude trends in drinking behavior. Mixed-effects models were used to study the consumption of total alcohol, wine, beer and spirits/other alcoholic beverages in relation to birth cohort, socio-demographic, lifestyle and health factors. We observed a decreasing trend of alcohol intake as age increased, consistent for total alcohol consumption and the three types of beverages. Older birth cohorts had lower initial total alcohol consumption (8 vs. 10 g/day) and steeper decline in wine, spirits/other alcoholic beverages and total alcohol consumption compared to younger cohorts. Higher education and smoking at baseline had a positive association with longitudinal total alcohol consumption, up to +30% (vs. low education) and more than +25% (vs. non-smoking) respectively, whereas female gender, obesity, history of heart attack, diabetes, peptic ulcer and high blood pressure at baseline had a negative association of −85%, −25%, −16%, −37%, −22% and −24% respectively. Alcohol consumption changed over age with different trends among the studied subgroups and types of alcohol, suggesting targeted monitoring of alcohol consumption.

## 1. Introduction

Some alcohol consumption patterns have been shown to be protective against various cardiovascular events [1] while at the same time high alcohol intake is a risk factor for various non-communicable chronic diseases, including cardiovascular disease, diabetes [2] and several types of cancer [3,4]. Given the important impact of alcohol intake on public health, the World Health Organization (WHO) emphasizes the value of close monitoring of longitudinal drinking patterns and their determinants across countries. Interestingly, the 2019 WHO European Region report on alcohol consumption is alarming for Europe, where the per capita alcohol intake varies widely by country but is still the highest in the world [5]. In this setting, longitudinal data is required to monitor continually changes in drinking behavior in the European region and guide related policies.

Identifying variables determining the amount of alcohol consumption is important in understanding their relationship with long-term drinking patterns. Older age, for example, has been associated with a decrease in alcohol consumption [6,7] or stable [8] drinking trends over the life course [6,9]. Other demographic predictors including sex [6,10,11], educational level [6], smoking habits and the onset of chronic conditions [12] have also been associated with changes in the longitudinal drinking behavior. Culture [13], religion/religiosity [14] and ethnicity [15] are also factors associated with alcohol consumption. Despite the important effect of alcohol use on public health, research on the predictors of longitudinal drinking behavior yielded conflicting findings [12,16,17]. Conflicting findings could potentially be attributed to a predictor being associated in a different way with different types of alcohol. The association between the predictor and total alcohol may then give conflicting results if there are differences in the proportion of total alcohol derived from different alcohol categories. In addition, most cohort studies in this area assess participants’ alcohol intake at the time of entry into a cohort and provide no information during follow-up [18]. Additional limitations are related to the study sample size, the restricted follow-up time, or narrow age ranges [5,19,20,21].

Heterogeneity has also been observed in statistical approaches used in cohort studies to describe alcohol consumption over time. Some studies summarized alcohol consumption by estimating the average participant intake per day [22] over the follow-up period or over lifetime [1]. Exposure to alcohol has also been defined by qualitative measures and specifically by classifying participants into drinking categories, including abstainers, former, binge or occasional drinkers [17]. Other studies detected trends in drinking behavior using alcohol consumption trajectories [16], while some others used statistical models to describe and predict individual alcohol consumption and change in consumption over time [6,23].

The objective of our study is to describe the long-term changes in alcohol consumption (wine, beer, spirits) in the EPIC-Greece cohort participants, with data from 1994 up to 2011, and their relation with various sociodemographic factors and health determinants, including age, birth cohort, gender, BMI, medical history at baseline, smoking and physical activity, separately assessing the age and cohort effects. By scaling up the effects of several sociodemographic factors, we aim to provide guidance to policy-makers to design and implement national strategies towards reducing the harmful use of alcohol.

## 2. Materials and Methods

### 2.1. The EPIC-Greece Cohort

EPIC is a multicenter prospective cohort study, in 10 European countries investigating the association of several risk factors, with a special focus on the study of the association of diet with cancer and other health outcomes [24]. Many publications of this major study have contributed to our knowledge on nutrition and health [25,26,27]. The EPIC-Greece cohort consists of 28,572 adult volunteers recruited between 1994 and 1997. At enrollment, detailed information on the participants’ demographic and socioeconomic characteristics, physical activity, anthropometric measurements, medical and reproductive history was recorded. Dietary intake at baseline was interviewer-administered via a detailed semi-quantitative food frequency questionnaire (FFQ) [28].

### 2.2. Follow-Up

After the initial enrollment, between 1994 and 1997, 26,579 participants were followed-up every 3–4 years in order to record changes in lifestyle and the incidence of various health outcomes. The follow-up of participants between 1997 and 2011 was conducted mainly by telephone interviewer-administered questionnaires (92.34% of the interviews) and to a small extend by mail (7.15%) or by personal interviews (0.51%). When the participant was not found, information on vital status was obtained by the next of kin. Information on the occurrence of cancer and cardiovascular diseases was confirmed by hospital records.

### 2.3. The Alcohol Follow-Up

The follow-up questionnaires included questions regarding the consumption of wine, beer and spirits/other beverages. For each type of these alcoholic drinks there were three pre-specified categories: “No consumption (None)”, “Occasionally” and “More than 1 glass per week”. Participants answering “More than 1 glass per week” had to specify how many glasses per week they are consuming on average. In order to have a measure of glasses of consumption of each type of drink for each participant, the decision was made to assign a quantity of 0.5 glasses per week for participants responding “Occasionally” during an interview.

### 2.4. Computation of Frequency and Quantity of Wine Consumption

During the early years of the follow-up period (1997–2004), information on wine consumption was collected via questions on total wine consumption while from 2005 onwards, separate questions were asked for four wine sub-categories namely, red wine, retsina (traditional resin enriched white wine in Greece), white wine non-resinated and rose wine. As focus is given in the total wine consumed, the data on consumption of the different wine subtypes were added based on the number of glasses of each subtype of wine. Thus, for the period 2005–2011, the amount of total wine consumption was estimated as the sum of the consumptions of the wine subtypes. The frequency variable with the three categories “No consumption (None)”, “Occasionally” and “More than 1 glass per week” was also reconstructed from the total quantity of wine, e.g., if the total quantity was less than 1 glass per week but not zero, then the category “Occasional drinker” was assigned regarding total wine consumption.

### 2.5. Computation of Total Alcohol Intake

Klipstein-Grobusch et al. [29] studied the trends of alcoholic beverages consumption and ethanol intake for the participants from all countries included in the EPIC study. Based on the 24-h dietary recalls they derived a table of type of alcohol, country, and gender specific correspondence between glasses of alcoholic beverages and alcohol intake (grams/day). We used the Greece—Specific correspondence to convert the glasses of wine, beer, and spirits/other alcoholic beverages per week into grams/day and then summed over them. The correspondence values are 12.5 g of ethanol per glass of wine for men and 9.1 g for women, 7.2 g of ethanol per glass of beer for men, and 5.7 g for women and 24.5 g of ethanol per glass of beer for men and 16 g for women.

### 2.6. Demographic Variables

The aim of the study is to investigate the relation of several socio-demographic, lifestyle, and health factors with long-term alcohol consumption as well as to model the aging and cohort effect. The demographic variables of interest in our study are age at baseline, gender, level of education, retirement, and marital status. The lifestyle factors used are body mass index (BMI) and smoking status at baseline. The participants are separated into three groups according to BMI value: <25 (normal weight), 25–30 (overweight) and >30 (obese). The health factors of interest are the history of heart attack, diabetes, peptic ulcer, high blood pressure and hypercholesterolemia as measured at baseline.

### 2.7. Study Sample

The EPIC study is a dynamic, continuous process that started in 1990. All subjects gave their informed consent for inclusion before they participated in the study. The study was conducted in accordance with the Declaration of Helsinki. In the present analysis we include alcohol data (baseline and follow-up) until 2011. Within a period of 14–16 years, each participant was interviewed approximately every 3–4 years. The baseline period was in 1994–1997, the first follow-up period was in 1997–2002, the second in 2002–2007 and the third was in 2007–2011.

### 2.8. Statistical Analysis

Stata software version 11 was used [30] for the derivation of frequency tables, summary statistics, figures as well as for the application of linear mixed models.

A mixed-effects linear regression model with random intercept and random slope for age at interview that includes birth cohort and gender was used in order to observe the overall changes in consumption of wine, beer, spirits/other alcoholic beverages and total alcohol over age for the different birth cohorts for men and women. The basic model included linear and quadratic terms for age at interview and interactions with the gender and birth cohort categorical covariates.

The correlation between the demographic (gender, education level, marital status, retirement), lifestyle (BMI, smoking) and health status factors (heart attack, diabetes, peptic ulcer, high blood pressure and hypercholesterolemia) with the longitudinal consumption of wine, beer and spirits/other beverages and total alcohol consumption (grams/day) was studied by including the aforementioned factors in model M_0_ as well as interaction terms with the linear term of the age at interview covariate.

As the distributions of the dependent variables are highly skewed towards zero with many non-consumers, we added a minimum quantity of 0.1 to the non-consumers and use the logarithm of the consumptions as the response variables in the models. In order to interpret the results in the normal scale, we present the exponentiated predictions and coefficients of the models. In the exponentiated context, a coefficient of 1 signifies no change in the response variable (e.g., consumption of wine), a coefficient of <1 signifies decreased consumption (e.g., a coefficient of 0.8 signifies 20% reduction) and a coefficient of >1 signifies increased consumption. Due to the complexity of the effect of time (quadratic) in the model and the interactions of the sociodemographic and lifestyle factors with age at interview, average marginal effects for different age at interview time points are used in order to give a summary of the relation of these factors with alcohol consumption.

The analyses included 22,721 individuals (85.5% of the sample) who have at least three interviews (Baseline interview and two follow up interviews) as we observed that participants who stayed less time in the cohort (fewer interviews), tended to report higher alcohol consumption, thus potentially influencing the analysis due to a reverse survivorship bias effect. The models were tested for inclusion or not of random slope, inclusion, or not of quadratic effect of the fixed main effect of time and interactions between the covariates. The interaction terms were kept even when not statistically significant. An initial significance level of a = 0.05 was used for all statistical analyses. Each participant contributes to the models with rows equal to the number of interviews that he/she has completed. Missing values for a particular type of alcoholic beverage are easily handled by the linear mixed models by not including the response for the specific row of the participant. The main source of loss to follow up is inability to get in further contact with the participant due to change in contact details or death.

## 3. Results

**Figure 1:**
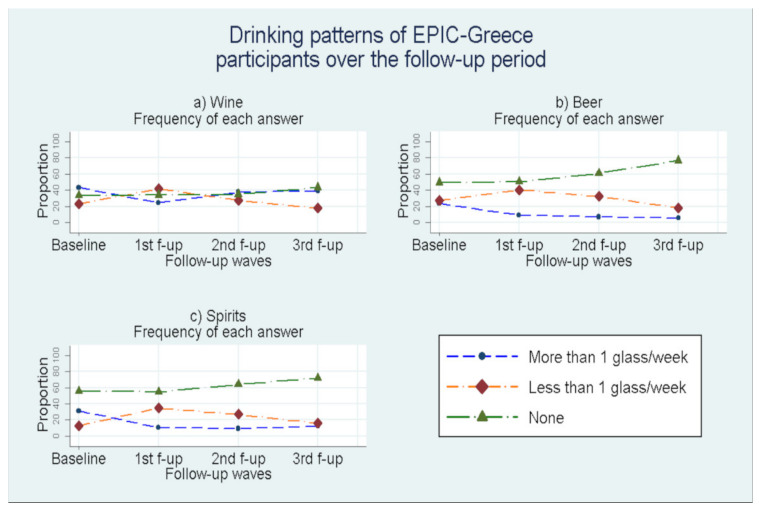
Line plots of frequencies of consumption for wine, beer and spirits/other alcoholic beverages at the baseline and the follow up periods (“None”, “Less than 1 glass/week”, “More than 1 glass/week”).

**Figure 2:**
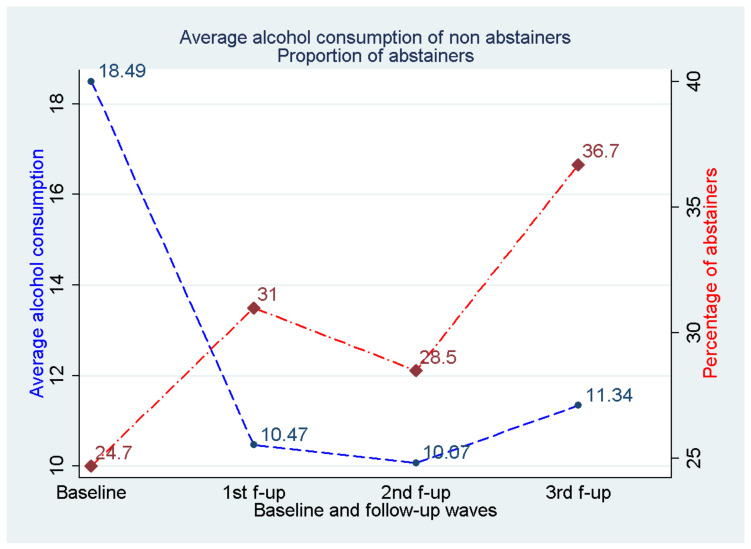
Average alcohol consumption (grams/day) from all sources for non-abstainers and proportion (%) of abstainers across time.

**Figure 3:**
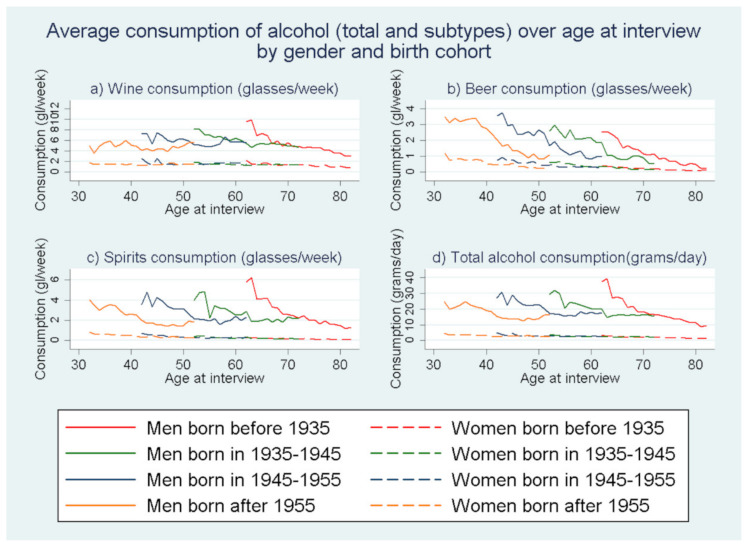
Line plots of average annual consumption for wine, beer, spirits/other alcoholic beverages (glasses/week) and total alcohol consumption (grams/day) for men and women of different birth cohorts.

Overall, 28,572 individuals (11,953 men and 16,619 women) were recruited in EPIC-Greece study from which 26,579 (10,943 men and 15,645 women) took part in the follow-up process. In specific, 24,352 participants took part in the first follow-up period (1 January 1997–14 November 2002), 22,220 participants took part in the second follow-up period (15 November 2002–24 June 2007), and 18,452 participants took part at the 3rd (25 June 2007–03 May 2011). The majority of the participants filled three follow-up questionnaires (15,969—60.08%) while 6752 (25.40%) participants filled 2 follow up questionnaires 3848 (14.47%) participants filled only one follow-up questionnaire and 10 participants filled four follow-up questionnaires until November 2011. From the 28,572 baseline participants, 1993 did not participate in any follow-up round. The frequencies of answers “No consumption (None)”, “Occasionally”, “More than 1 glass per week” regarding the alcoholic drinks consumption (wine, beer, spirits/other alcoholic beverages) are presented for the baseline period over the levels of the baseline demographic, lifestyle and health status factors (Table 1). Figure 1 gives the frequency (%) for each of the aforementioned drinking patterns over the study period (baseline and follow-up waves) for the three types of alcoholic beverages. Figure 2 depicts the average total alcohol consumption across the study period for the non-abstainers. Figure 3 contains line plots for the annual average consumption among men and women for four birth cohorts.

We observed an increase in the percentage of occasional drinkers and a decrease in the percentage of systematic drinkers in the first follow-up wave (Figure 1a). However, during the subsequent follow-up waves the trend was reversed, with an increase in those reporting systematic wine consumption and decrease in those reporting occasional consumption. Wine non-consumers presented a small but steady increase from 30% at baseline to 40% during the 3rd follow-up wave. Figure 1b depicts a different trend for drinking behavior of beer over the follow-up time, with the percentage of non-consumers increasing a great deal over follow-up (from 50% at baseline to 80% during the 3rd follow-up wave). Similarly, the percentage of systematic consumers followed an important decrease from 20% at baseline to 2–3% during the 3rd follow-up wave. The percentage of occasional beer consumers increased substantially in the 1st follow-up wave only to decrease back to the baseline levels during the 3rd follow-up wave. A pattern similar to that of Figure 1b can be discerned from Figure 1c for spirits and other alcoholic beverages, with the percentage of non-consumers increasing significantly over time (from 60% at baseline to 75% during the 3rd follow-up wave). The percentage of systematic consumers dropped significantly over time (from almost 40% at baseline to less than 10% during the 3rd follow-up wave). As in the case of beer, here as well the percentage of occasional spirits consumers increased substantially in the 1st follow-up wave only to decrease back to the baseline levels during the 3rd follow-up wave. Figure 2 is a composite graph depicting simultaneously the percentage of abstainers from any type of alcohol (red line) and the average total alcohol consumption (grams/day) for non-abstainers. It can be seen that the percentage of abstainers from all types of alcohol increased over time (from 26.7 to 41.4 during the 3rd period). At the same time, the average total daily consumption dropped from 16.7 g/day at baseline to almost 10 g/day during the rest of the follow-up.

**Figure A1:**
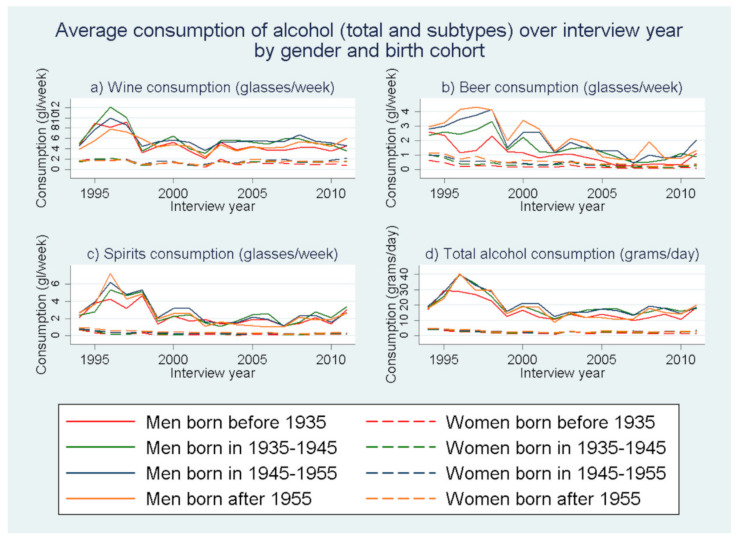
Line plots of average annual consumption for wine, beer, spirits/other alcoholic beverages (glasses/week) and total alcohol consumption (grams/day) for men and women of different birth cohorts over interview year.

**Figure A2:**
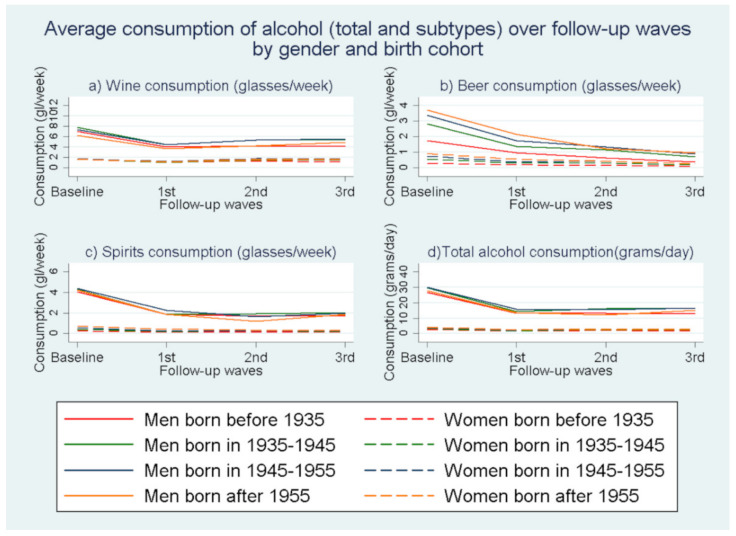
Line plots of average annual consumption for wine, beer, spirits/other alcoholic beverages (glasses/week) and total alcohol consumption (grams/day) for men and women of different birth cohorts over the baseline period and the follow-up waves.

Figure 3 depicts the change in the average annual consumption over the different types of alcoholic beverages and total alcohol with increasing age at interview. Figure A1 and Figure A2 of Appendix A depict the average annual consumptions over calendar year and follow-up waves. According to Figure 3a, the older the birth cohort, a steeper the decline in the average annual consumption of wine was observed. Regarding average annual beer consumption (Figure 3b), the younger cohorts showed to have a larger beer consumption but the rate of decline was similar between all birth cohorts. The rates of decline in the average annual consumption of spirits/other alcoholic beverages (Figure 3c) and total alcohol consumption (Figure 3d) was similar between the different birth cohorts, but with a slightly steeper decline for the oldest birth cohort.

**Figure 4:**
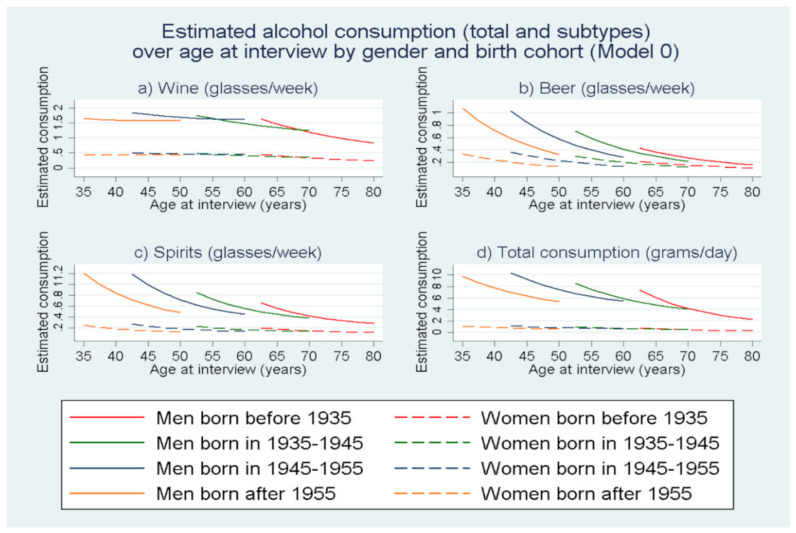
Line plots of estimated consumption for wine, beer and spirits/other alcoholic beverages over age at interview for four birth cohorts for men and women, based on the linear mixed model Model 0 (M_0_).

Figure 4 shows the estimates from the linear mixed model M_0_ for the consumption of wine, beer, spirits/other alcoholic beverages as well as total alcohol consumption by age at interview and birth cohort. The estimates of consumption by age at interview are given for four different birth cohorts (participants born before 1935, 1935–1945, 1945–1955 and after 1955). The estimated consumption of all three types of alcohol were significantly higher in men compared to women. Women had low alcohol consumptions throughout the cohort and even presented a further small decrease in beer consumption. Wine consumption presented a small decline over age with the decline getting steeper for the older birth cohorts (Figure 4a). Regarding beer and spirits/other alcoholic beverages consumption for men (Figure 4b,c) there was a gradual decline in consumption, as the participants grow older. The younger cohorts (1945–1955 and after 1955) entered the follow-up period with higher initial consumption compared to the older cohorts but also presented a steeper decline of beer and spirits/other alcoholic beverages consumption with increasing age when we compare the age intervals where the estimates for the different birth cohorts overlap (ages 43–50, 53–60, 63–70). The total alcohol consumption presented a similar, mild rate of decline among the birth cohorts, being less steep as age progresses.

Table 2 contains the exponentiated coefficient estimates of the models for logarithm of wine, beer, spirits/other beverages and total alcohol consumption (M1), their confidence intervals and their statistical significance on the 0.05 significance level. Due to the complexity of the regression model that emerges from the quadratic terms for time in cohort and age at baseline as well as the interactions between age and the rest of the independent variables, the straightforward interpretation of the overall relation between a covariate and the estimated alcohol consumptions become taxing.

**Figure 5:**
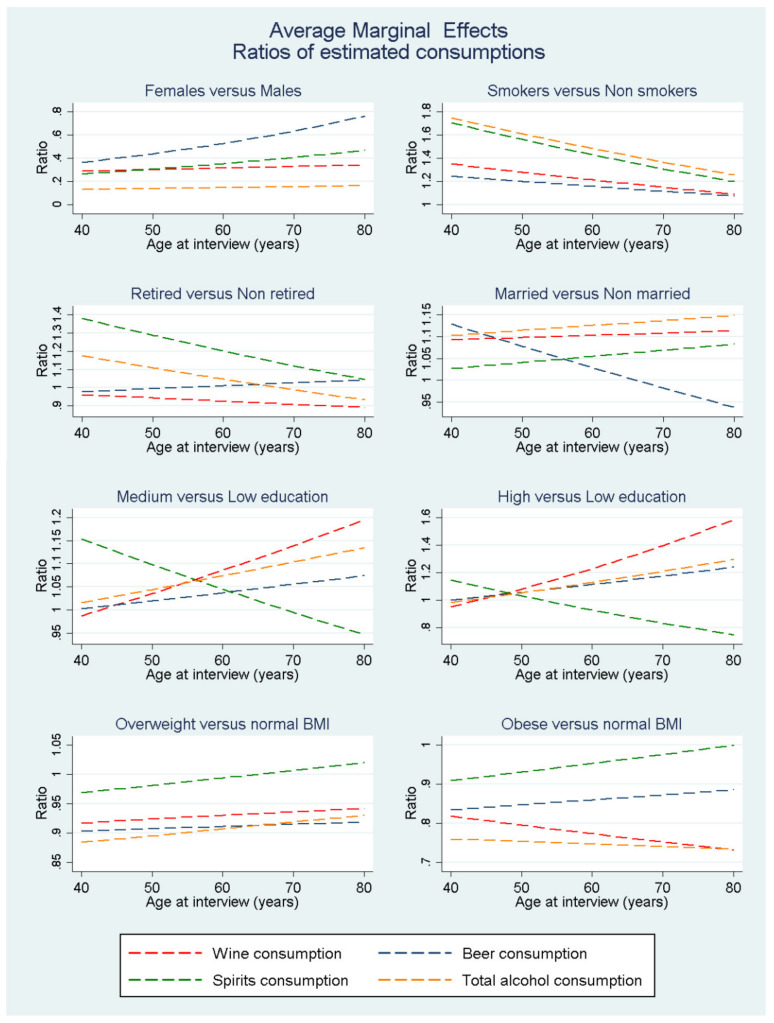
Average marginal effects of socio-demographic and lifestyle factors on wine, beer, spirits/other alcoholic beverages and total alcohol consumption based on model of Table 2.

In this paragraph, we provide a general interpretation of the covariate effects of Table 2. However, for a better grasp of the overall relation between the factors under study and alcohol consumption, we propose Figure 4 (crude model) for the depiction of the change of alcohol consumption over age for the different birth cohorts. For the sociodemographic and lifestyle factors we propose Figure 5 and Table A1 (Appendix A) where the overall effects (average marginal effects) for each factor are presented over age. Only the relation of the health factors with the alcohol consumption variables can be easily interpreted as we did not include interactions with age for those covariates. We observe that, as age at interview increased (5-year steps) the linear term of the variable indicates an overall reduction of 20–32% for total alcohol and the alcohol sub-categories. However, the quadratic term for age at interview is over 1, signifies that this effect weakened over age. Another observation is that the main effects of the birth cohort covariates suggest that younger cohorts appeared to consume less compared to older birth cohorts given a specific age at interview. The age–birth cohort interaction terms indicate that this effect weakened over age. The interpretation of the results related to birth cohorts is proper under overlapping age ranges between the different birth cohorts to avoid extrapolation. For example, for age at interview equal to 50, the spirits/other alcoholic beverages consumption was lower for the younger birth cohort (after 1955) compared to the previous one (1945–1955). However, if we compare both these cohorts at the start of their respective follow-up, their initial spirits consumption would be about the same. Being female, and being overweight or obese had main covariate effects that suggest lower alcohol consumption compared to being male and normal weight respectively. Being of medium or high education, being a smoker and being married have main covariate effects that suggested a statistically significant increase in consumption when compared to low education, being a non-smoker and not being married respectively. A trend can be observed for medium and high education, the higher the education level, the higher the wine consumption compared to individuals with low education. The same was observed for beer consumption, albeit to a lesser extent. Having retired suggested decreased wine consumption and increased spirits consumption. Participants who had a history of heart attack at baseline consume 11% less wine, 10% less beer, 8% spirits/other beverages and 16% less total daily alcohol compared to heart attack free individuals. However only the estimate for beer and total alcohol seemed to be significant at the 0.05 significance level. Participants with a history of diabetes consumed 31% less wine, 17% less beer, 16% less spirits/other beverages and 36% less total daily alcohol compared to diabetes free participants. Participants with history of high blood pressure at baseline consumed 25% less wine, 6% less beer, 4% less spirits/other beverages and a total of 24% less total daily alcohol compared with participants without a history of high blood pressure at baseline. The factor of high blood cholesterol at baseline did not present any significant correlation with alcohol consumption. Finally, participants with a history of peptic ulcer consume 17% less wine, 5% less beer, 12% less spirits/other beverages and a total of 22% less total daily alcohol compared to participants without a history of peptic ulcer.

Figure 5 and Table A1 of the Appendix A show the overall effects of socio-demographic and lifestyle factors on wine, beer, spirits and total alcohol consumption based on model M_1_. Being female vs. being male showed smaller alcohol consumption for females ranging from 60 to 70% reduced consumption in wine and spirits/other beverages, a 30–62% and more than 80% reduced consumption in total alcohol. Only the beer consumption difference between men and women decreased over age at interview, a result of the decreased beer consumption in older men rather than an increase in consumption for women. The overall association of smoking vs. being non-smoker with alcohol consumption was quite high, with smokers consuming almost 80% more spirits/other alcoholic beverages and total alcohol at 40 years of age at interview a surplus that declined over time reaching 20–30%. Smokers also tended to consume more wine and beer compared to non-smokers compared to the non-smokers, with smokers consuming almost 35% more wine and 25% total alcohol at 40 years of age at interview, an excess consumption that declines over time reaching under 10% surplus at the 3rd follow-up.

Participants who were already retired at baseline seemed to consume approximately the same amount of wine and beer compared to not retired participants, with difference ranging from −3% to −10% for wine and +13% to −6% for beer over age. Retired participants seemed to consume on average 40% more spirits/other alcoholic beverages and 20% more alcohol in total compared to non-retired individuals, but with the progression of age, this difference was minimized. Being married at baseline vs. not being married was associated with a slightly higher wine and total alcohol consumption for the married participants (around 10%). The average spirits/other alcoholic beverage consumption seemed similar over age between the two groups while the beer consumption of married participants seemed to decrease over time in comparison to the non-married participants, going from +15% at 40 years of age to −5% at 80 years of age. Participants with medium or high education seemed to have on average, a much higher wine consumption, a difference that increased as age progressed and reached +20% for medium education and 60% for high education level individuals at the age of 80, indicating a positive trend over higher educational levels. Participants with medium or high education also presented a slightly higher beer and total alcohol consumption compared to participants of low education level (up to +20% for total alcohol at age 80). The opposite was observed for spirits/other alcoholic beverages consumption with medium and high education level individuals having on average, a higher consumption at younger ages compared to the low education level individuals but they ended up consuming less than them as age progresses (+14% at age 40 to −25% at age 80 for the high education participants).

Regarding the average marginal effect of the BMI groups, overweight participants appeared to consume on average, slightly less wine, beer and total alcohol compared to the normal weight participants (around −10%) and approximately the same amount of spirits/other alcoholic beverages. Obese participants consumed even less wine, beer and total alcohol compared to normal weight participants with a range of −12% to −27% that seemed quite steady as age progressed. Regarding spirits/other alcoholic beverages the consumption between normal weight and obese participants was quite similar, ranging from a difference of −10% to 0% over age.

## 4. Discussion

This study aimed to assess the long-term changes in alcohol consumption (wine, beer, spirits/other alcoholic beverages and total alcohol) of the Greek EPIC cohort participants over age and study its association with socio-demographic, lifestyle and health factors measured at baseline.

An increase is observed in the frequency of alcohol abstainers as well as a reduction of the average consumption over time. These results agree with McEvoy et al. [8] and Molander et al. [7] who reported a decline in average alcohol consumption over time and Bray et al. [16] reporting a strong increase in non-drinkers with increasing age.

Alcohol consumption decreased as age progressed. Beer, spirits/other alcoholic beverages and total alcohol consumption tended to decrease with age while wine consumption tended to have a more linear decrease with older cohorts presenting steeper decreases over age. Declining alcohol consumption as age progresses is also reported by Moore et al. [6], Platt et al. [31], Levenson et al. [32] and Britton et al. [33]. One potential explanation for this decline could be the emergence of health issues and medication indications as age progresses that require reduction or abstinence from alcohol.

We also observed that younger birth cohorts started the follow-up period with higher average consumption of total alcohol, beer, spirits/other alcoholic beverages and similar wine consumption compared to the older birth cohorts. In addition, older birth cohorts tended to present more steep decline in alcohol consumption (except beer) than younger cohorts when we compared the overlapping age intervals between the different birth cohorts (ages 43–50, 53–60, 63–70). Results from Levenson et al. [32] also show that younger birth cohorts had increased alcohol consumption.

In all cases, women presented a much lower alcohol consumption over age at interview compared to men, being in accordance with relevant literature [6,7,31]. However, it is interesting to notice that the gap in beer consumption between men and women seemed to be closing as time of follow-up progressed.

Among the rest of the socio-demographic factors and the lifestyle factors included in the statistical models, smoking was related with a higher alcohol consumption agreeing with the relevant findings from Moore et al. [6]. This was observed primarily for spirits/other alcoholic beverages and total alcohol but with the trend weakening over age. Higher levels of education were related with a higher wine, beer and total alcohol consumption [6,7,19,31] with the trend intensifying over age. Being overweight or obese at baseline was related with a decreased consumption of wine, beer and total alcohol consumption. Having retired was related with an increased spirits/other alcoholic beverages and total alcohol consumption that weakened over age. A variable similar to being retired or not exists in Moore et al. (working/not working) that does not present any relation with alcohol consumption. Being married was found to be related with slightly increased wine and total alcohol consumption agreeing with part of the literature [6,19] indicating higher alcohol consumption among married individuals. However, we also observed that participants who were married at baseline presented a more rapid decline of spirits/other alcoholic beverages over age compared to not being married, agreeing with results from other studies regarding alcohol consumption [31,34].

Among the health conditions considered, participants with diabetes and history of heart attack at baseline had significantly smaller alcohol consumption (over all three types of alcohol plus total alcohol) compared to compared to healthy individuals. Participants with history of peptic ulcer at baseline presented a lower consumption compared to healthy participants, primarily for wine, spirits and total alcohol. Participants with high blood pressure at baseline appeared to consume significantly less wine and total alcohol than healthy. Finally, having high blood cholesterol at baseline did not show any relation with alcohol consumption. Relevant studies from literature report that individuals in good health tended to have higher levels of alcohol consumption [7,19,31]. 

There are different views on the use of alcohol and its relation to health. Many studies show that moderate alcohol consumption, especially wine, can be beneficial for health within the framework of a healthy diet [35,36,37,38,39]. Other studies and guidelines associate any alcohol use with a certain health risk [2,3,4,40], stating that even low alcohol consumption carries a certain risk. The present study is purely descriptive and aims to provide information on how alcohol consumption is associated with age, gender, follow- up period and other factors for the participants of the EPIC- Greece cohort.

This study has a number of strengths and several limitations. EPIC-Greece is a large cohort study, including more than 28,500 participants aging from 20 to 86 years of age. The large majority of the participants had at least three interviews (baseline plus two follow-up interviews). The participants were recruited from all over the country, thus covering a broad range of socio-demographic factors [27,41]. In contrast to other studies that focus only on total alcohol consumption, our study includes results on the consumption of the three basic alcohol subtypes (wine, beer and spirits/other alcoholic beverages) as well as for total alcohol. Another strong point of our study is that we allow for flexible modelling of age using quadratic terms in our models. We also include interactions between the socio-demographic and lifestyle factors with age, in order to capture properly time varying effects on alcohol consumption.

One of the limitations of the study is the presence of misreporting because of recall bias and other perception fallacies. Another issue is that not all participants participated in each one of the follow-up periods. Additionally, albeit a large sample, the EPIC-Greece cohort was not designed to be a representative sample of the Greek population. Therefore, it would be preferable to avoid generalizing the trends observed in this study to the overall population.

The age, period, and cohort (APC) effects identification problem, as described in detail by Bell and Jones [42], is the inability of identifying and disentangling the APC effects without making the assumption that at least one of them has zero effect. The models used in the statistical analysis of this study can be considered valid in regards with the interpretation of the effect of age and the birth cohorts on the change of alcohol consumption provided a lack of period effect on the alcohol consumption is assumed. That means that we assume there was no considerable change in the overall alcohol consumption between 1994 and 2011 that affected all age and cohort groups simultaneously, for example, state regulations or socioeconomic changes. Data from WHO on per capita alcohol use in Greece show a decrease from 12 L in 1990 to 10 L in 2011 [43]. However, we cannot distinguish if this observation is due to a period effect or due to the aging of the population (from median age of 36.5 in 1995 to 41.1 in 2010) [44]. In Greece there have been campaigns and recommendations from health practitioners for the reduction of alcohol consumption since the early nineties, but no particular alcohol regulation has been implemented. The health recommendations and campaigns may have played a role in the reduction of alcohol use over the study period but due to the limitation imposed by the APC identification problem, we assume the period effect to be zero. In addition, a big socioeconomic change that was observed in 2010 due to the financial crisis in the public sector is near the end of the analysis period. A study conducted in Italy studying APC effects during a similar time period (1997–2002) did not indicate any particular period effects in alcohol use [45]. A similar study conducted for Great Britain shows no period effects for men but significant period effects for women (increase in alcohol consumption 1990 to 1994 onwards) [46].

## 5. Conclusions

For the participants of the EPIC-Greece cohort, age progression is related with a gradual decrease of beer and spirits/other alcoholic beverages and total alcohol consumption with the exception of wine, which shows a decline only for the oldest birth cohort. Younger birth cohorts present higher initial (start of follow-up) alcohol consumption while older cohorts present a slightly steeper decline of alcohol consumption over age for age intervals where the estimations for the different birth cohorts overlap. In all cases, men have a much higher alcohol consumption compared to women. The change in alcohol consumption does not appear the same over age among the different sociodemographic and lifestyle subgroups, indicating that targeted monitoring of alcohol consumption, and health policy responses and strategies on specific subpopulation groups should be encouraged. With alcohol consumption being interwoven with human health, both on a population and an individual level, further studies should be conducted regarding the trends of alcohol consumption, factors related to it, while also taking into account the different types of alcohol.

## Figures and Tables

**Table 1:** Number and percentage (%) of abstainers, occasional and systematic drinkers by socio-demographic, lifestyle, and health factors of interest at baseline. The EPIC-Greece study.

		Overall	Abstainers No Consumption	Occasional—Less Than 1 Glass/Week			Systematic—More Than 1 Glass/Week		
				Wine	Beer	Spirits	Wine	Beer	Spirits
Age Mean (±SD)		53 (±12.7)	56.7 (±12.7)	51.6 (±12.4)	51.5 (±12.1)	50.6 (±12)	52 (±12.3)	48.6 (±11.7)	49.8 (±12.23)
Gender N (%)	Males	11,954 (41.8)	1397 (18.6)	2211 (33.7)	3187 (41.8)	1639 (44.4)	7261 (59.9)	4422 (66.9)	6432 (73.5)
	Females	16,618 (58.2)	6101 (81.4)	4344 (66.3)	4434 (58.2)	2051 (55.6)	4853 (40.1)	2185 (33.1)	2324 (26.5)
Education N (%)	Low	5884 (21)	2372 (31.6)	1168 (17.8)	1201 (15.8)	491 (13.3)	1974 (16.3)	731 (11.1)	1018 (11.6)
	Medium	17,330 (61.8)	4411 (58.8)	3970 (60.6)	4826 (63.3)	2366 (64.2)	7810 (64.5)	4252 (64.4)	5654 (64.6)
	High	4816 (17.2)	713 (9.5)	1417 (21.6)	1592 (20.9)	832 (22.6)	2327 (19.2)	1624 (24.6)	2082 (23.8)
Marital status N (%)	Single	4406 (15.7)	1482 (19.8)	1082 (16.5)	1143 (15)	526 (14.3)	1556 (12.9)	882 (13.4)	1184 (13.5)
	Married	23,612 (84.3)	6008 (80.2)	5470 (83.5)	6475 (85)	3162 (85.7)	10552(87.1)	5723 (86.6)	7567 (86.5)
Retired N (%)	No	22,397 (78.4)	5355 (71.4)	5328 (81.3)	6274 (82.3)	3038 (82.4)	9664 (79.8)	5703 (86.3)	7104 (81.1)
	Yes	6175 (21.6)	2143 (28.6)	1227 (18.7)	1347 (17.7)	652 (17.7)	2450 (20.2)	904 (13.7)	1652(18.9)
BMI group N (%)	Normal	6263 (21.9)	1420 (18.9)	1597 (24.4)	1780 (23.4)	922 (25)	2745 (22.7)	1715 (26)	2062 (23.5)
	Overweight	12,139 (42.5)	2834 (37.8)	2765 (42.2)	3323 (43.6)	1613 (43.7)	5723 (47.2)	3144 (47.6)	4308 (49.2)
	Obese	10,170 (35.6)	3244 (43.3)	2193 (33.5)	2518 (33)	1155 (31.3)	3646 (30.1)	1748 (26.5)	2386 (27.2)
Smoking at baseline N (%)	No	15,369 (53.9)	5515 (73.6)	3765 (57.5)	4015 (52.8)	1830 (49.7)	5119 (42.3)	2295 (34.8)	2481 (28.4)
	Yes	13,168 (46.1)	1978 (26.4)	2778 (42.5)	3589 (47.2)	1851 (50.3)	6981 (57.7)	4306 (65.2)	6266 (71.6)
History of heart attack at baseline N (%)	No	27,492 (98.1)	7336 (97.9)	6466 (98.6)	7496 (98.4)	3618 (98.1)	11854(97.9)	6532 (98.9)	8564 (97.8)
	Yes	538 (1.9)	160 (2.1)	89 (1.4)	123 (1.6)	71 (1.9)	257 (2.1)	75 (1.1)	190 (2.2)
History of diabetes at baseline N (%)	No	26,041 (92.9)	6634 (88.5)	6170 (94.1)	7224 (94.8)	3500 (94.9)	11491(94.9)	6424 (97.2)	8379 (95.7)
	Yes	1989 (7.1)	862 (11.5)	385 (5.9)	395 (5.2)	189 (5.1)	620 (5.1)	183 (2.8)	375 (4.3)
History of peptic ulcer at baseline N (%)	No	26,749 (95.4)	7064 (94.2)	6314 (96.3)	7340 (96.3)	3535 (95.8)	11581(95.6)	6359 (96.2)	8390 (95.8)
	Yes	1281 (4.6)	432 (5.8)	241 (3.7)	279 (3.7)	154 (4.2)	530 (4.4)	248 (3.8)	364 (4.29)
History of high blood pressure at baseline N (%)	No	21,309 (76)	4800 (64)	5155 (78.6)	6100 (80.1)	3016 (81.8)	9953 (82.2)	5664 (85.7)	7337 (83.8)
	Yes	6721 (24)	2696 (36)	1400 (21.4)	1519 (19.9)	673 (18.2)	2158 (17.8)	943 (14.3)	1417 (16.2)
History of high blood cholesterol at baseline N (%)	No	21,178 (75.6)	5409 (72.2)	5007 (76.4)	5900 (77.4)	2871 (77.8)	9296 (76.8)	5310 (80.4)	6866 (78.4)
	Yes	6851 (24.4)	2087 (27.8)	1548 (23.6)	1719 (22.6)	818 (22.2)	2814 (23.2)	1297 (19.6)	1888 (21.6)

542 participants had missing values for education and the health variables. The same participants plus 12 more did not have marital data. 35 participants did not have smoking data.

**Table 2:** Age at interview, birth cohort, socio-demographic, lifestyle, health factors and predictions for wine, beer, spirits and total alcohol consumption.

	Wine ^a^	CI ^b^	Beer ^a^	CI ^b^	Spirits ^a^	CI ^b^	Total Alcohol ^a^	CI ^b^
Age at Interview ^c^ (5-year leaps)	0.799 *	(0.792–0.806)	0.733 *	(0.728–0.738)	0.721 *	(0.716–0.726)	0.675 *	(0.668–1.003)
Age at Interview squared	1.001 *	(1.001–1.002)	1.002 *	(1.002–1.002)	1.005 *	(1.005–1.006)	1.003 *	(1.003–0.641)
Cohort 1935–1945	0.699 *	(0.647–0.754)	0.789 *	(0.748–0.833)	0.726 *	(0.684–0.77)	0.589 *	(0.542–0.509)
Cohort 1945–1955	0.657 *	(0.606–0.712)	0.508 *	(0.481–0.538)	0.564 *	(0.53–0.601)	0.465 *	(0.426–0.376)
Cohort after 1955	0.624 *	(0.559–0.695)	0.289 *	(0.268–0.312)	0.42 *	(0.386–0.457)	0.334 *	(0.296–0.153)
Female	0.315 *	(0.303–0.328)	0.527 *	(0.514–0.54)	0.353 *	(0.343–0.364)	0.146 *	(0.139–1.146)
Medium education	1.086 *	(1.024–1.152)	1.037	(0.997–1.079)	1.045	(0.998–1.094)	1.074 *	(1.006–1.228)
High education	1.228 *	(1.138–1.324)	1.114 *	(1.061–1.17)	0.927 *	(0.875–0.982)	1.129 *	(1.039–0.954)
Overweight	0.93 *	(0.889–0.973)	0.911 *	(0.886–0.937)	0.994	(0.961–1.028)	0.907 *	(0.862–0.787)
Obese	0.773 *	(0.737–0.811)	0.86 *	(0.834–0.885)	0.953 *	(0.92–0.988)	0.746 *	(0.708–1.122)
Retired	0.926 *	(0.87–0.986)	1.011	(0.97–1.055)	1.201 *	(1.145–1.261)	1.047	(0.978–1.551)
Smoker	1.213 *	(1.165–1.262)	1.157 *	(1.129–1.186)	1.429 *	(1.387–1.472)	1.483 *	(1.418–1.187)
Married	1.103 *	(1.052–1.157)	1.029	(0.998–1.061)	1.055 *	(1.018–1.094)	1.126 *	(1.067–0.976)
Heart	0.885	(0.775–1.01)	0.897 *	(0.837–0.962)	0.917	(0.835–1.007)	0.842 *	(0.726–0.677)
Diabetes	0.689 *	(0.643–0.737)	0.825 *	(0.796–0.855)	0.84 *	(0.801–0.882)	0.627 *	(0.581–0.796)
HBP	0.755 *	(0.724–0.788)	0.944 *	(0.923–0.966)	0.965 *	(0.936–0.995)	0.76 *	(0.725–1.059)
HBC	1.022	(0.983–1.062)	0.981	(0.96–1.003)	0.987	(0.96–1.016)	1.014	(0.972–0.856)
Peptic	0.833 *	(0.77–0.901)	0.949 *	(0.907–0.992)	0.884 *	(0.834–0.937)	0.784 *	(0.718–1.044)
Age x Cohort 1935–1945	1.024 *	(1.016–1.031)	0.999	(0.994–1.005)	1.025 *	(1.019–1.031)	1.036 *	(1.028–1.067)
Age x Cohort 1945–1955	1.038 *	(1.027–1.05)	1	(0.991–1.008)	1.04 *	(1.031–1.049)	1.054 *	(1.042–1.08)
Age x Cohort after 1955	1.047 *	(1.031–1.063)	1.001	(0.989–1.013)	1.054 *	(1.041–1.066)	1.062 *	(1.045–1.008)
AgexFemale	1.004 *	(1.002–1.007)	1.019 *	(1.017–1.021)	1.014 *	(1.012–1.017)	1.005 *	(1.002–1.008)
Agex Medium education	1.005 *	(1–1.009)	1.002	(0.999–1.005)	0.995*	(0.992–0.998)	1.003	(0.998–1.013)
Agex High education	1.013 *	(1.007–1.018)	1.005 *	(1.002–1.009)	0.989*	(0.985–0.993)	1.007 *	(1.001–1.004)
AgexOverweight	1.001	(0.998–1.004)	1	(0.998–1.002)	1.001	(0.999–1.003)	1.001	(0.998–1.003)
AgexObese	0.997	(0.994–1)	1.001	(0.999–1.004)	1.002 *	(1–1.005)	0.999	(0.996–0.999)
AgexRetired	0.998	(0.994–1.003)	1.002	(0.999–1.005)	0.993 *	(0.99–0.996)	0.994 *	(0.989–0.995)
AgexSmoker	0.995 *	(0.992–0.997)	0.996 *	(0.995–0.998)	0.991 *	(0.989–0.993)	0.992	(0.989–1.004)
AgexMarital	1	(0.998–1.003)	0.995 *	(0.993–0.997)	1.001	(0.999–1.004)	1.001	(0.998–9.495)
Constant	1.997	(1.804–2.21)	0.52	(0.486–0.556)	0.59	(0.546–0.637)	8.485	(7.582–9.516)
Random int.	1.025		0.3171		0.567		1.283	
Random slope	0.0001		0.0002		0.00003		0.00025	
R. interecept- slope cov	0.0031		−0.0093		−0.0043		0.0046	
Log likelikhood	−150,398.47		−124,277.06		−129,615.7		−157,041.57	

^a^: The dependent variables of the models are were the log transformed consumptions log (consumption + 0.1). We add the 0.1 to include the abstainers. The coefficients presented on the table are the exponentiated parameter estimates. Coefficients with less than1 are associated with less drinking while coefficients > 1 with higher alcohol consumption. ^b^: Confidence interval, ^c^: Baseline is centered at age 60. * statistically significant on the 0.05 level.

## Data Availability

The data of the EPIC Greece cohort supporting the results of this study are available from Hellenic health Foundation. Restrictions apply to the availability of these data, which were used under license for this study.

## Appendix A

**Table A1:** Ratios of estimated consumption (Average marginal effects) for socio-demographic and lifestyle factors on wine, beer, spirits and total alcohol consumption based on model of Table 2 (M_1_).

**Marginal Effects** **Ratios**	**Wine**					**Beer**				
	**Age at Interview**					**Age at Interview**				
	**40**	**50**	**60**	**70**	**80**	**40**	**50**	**60**	**70**	**80**
Female vs. Male	0.289	0.302	0.315	0.329	0.343	0.364	0.438	0.527	0.634	0.764
Middle vs. Low education	0.987	1.035	1.086	1.139	1.195	1.002	1.02	1.037	1.056	1.074
High vs. Low education	0.951	1.08	1.228	1.395	1.585	1.001	1.056	1.114	1.176	1.241
Overweight vs. normal weight	0.917	0.924	0.93	0.936	0.942	0.903	0.907	0.911	0.915	0.918
Obese vs. normal weight	0.818	0.795	0.773	0.752	0.731	0.834	0.847	0.86	0.872	0.886
Smoking vs. non smoking	1.351	1.28	1.213	1.149	1.089	1.246	1.201	1.157	1.115	1.075
Retired vs. non retired	0.962	0.944	0.926	0.909	0.892	0.979	0.995	1.011	1.027	1.044
Married vs. not married	1.093	1.098	1.103	1.108	1.114	1.129	1.078	1.029	0.982	0.938
	**Spirits/Other Beverages**					**Total Alcohol Consumption**				
	**Age at Interview**					**Age at Interview**				
	**40**	**50**	**60**	**70**	**80**	**40**	**50**	**60**	**70**	**80**
Female vs. Male	0.265	0.306	0.353	0.407	0.47	0.131	0.138	0.146	0.154	0.162
Middle vs. Low education	1.154	1.098	1.045	0.994	0.946	1.016	1.044	1.074	1.104	1.135
High vs. Low education	1.148	1.032	0.927	0.833	0.749	0.984	1.054	1.129	1.21	1.296
Overweight vs. normal weight	0.969	0.981	0.994	1.007	1.02	0.884	0.895	0.907	0.919	0.93
Obese vs. normal weight	0.909	0.931	0.953	0.976	1	0.759	0.753	0.746	0.74	0.733
Smoking vs. non smoking	1.706	1.562	1.429	1.308	1.197	1.75	1.611	1.483	1.366	1.257
Retired vs. non retired	1.381	1.288	1.201	1.12	1.045	1.175	1.11	1.047	0.989	0.933
Married vs. not married	1.027	1.041	1.055	1.069	1.083	1.103	1.115	1.126	1.137	1.149
